# Integration of Rare Diseases into Social Services

**DOI:** 10.1186/1750-1172-9-S1-P11

**Published:** 2014-11-11

**Authors:** Raquel Castro, Dorica Dan

**Affiliations:** 1 European Organisation for Rare Diseases – EURORDIS, Paris, France; 2 Romanian National Alliance for Rare Diseases, Zalau, Romania; 3 Romanian Prader-Willi Association, Zalau, Romania; 4 EURORDIS, Paris, France; 5 European Commission Expert Group on Rare Diseases â€“ DG Health and Consumers, Brussels, Belgium

## Background

Social Services are instrumental to the empowerment of people living with rare diseases and to the improvement of their well-being and health. However, these services are still scarce and often not adapted to the needs of people living with rare diseases. Access to these services remains a challenge for patients and families affected by rare diseases.

## Objective

The European Committee of Experts on Rare Diseases Joint Action Work Package 6 - “Specialised Social Services and Integration of RDs into social services and policies” (enabled by EC Co-funding 20112201) - aims at giving more visibility to existing Specialised Social Services and good practices as well as advocating for the integration of people living with rare diseases in services not specific/exclusive to rare diseases, by working on training of social services providers.

## Method

In order to achieve the objectives above, the following methods have been used: mapping of Specialised Social Services available in Europe via a collection of contacts among EURORDIS members and network; collecting guiding principles for Specialised Social Services and for the Training of social services providers through the organisation multi-stakeholder workshops; compiling case study documents on expert existing services by organising country visits to expert services and applying a detailed questionnaire; advocating for these services via policy fact sheets.

## Results

**Figure 1:**
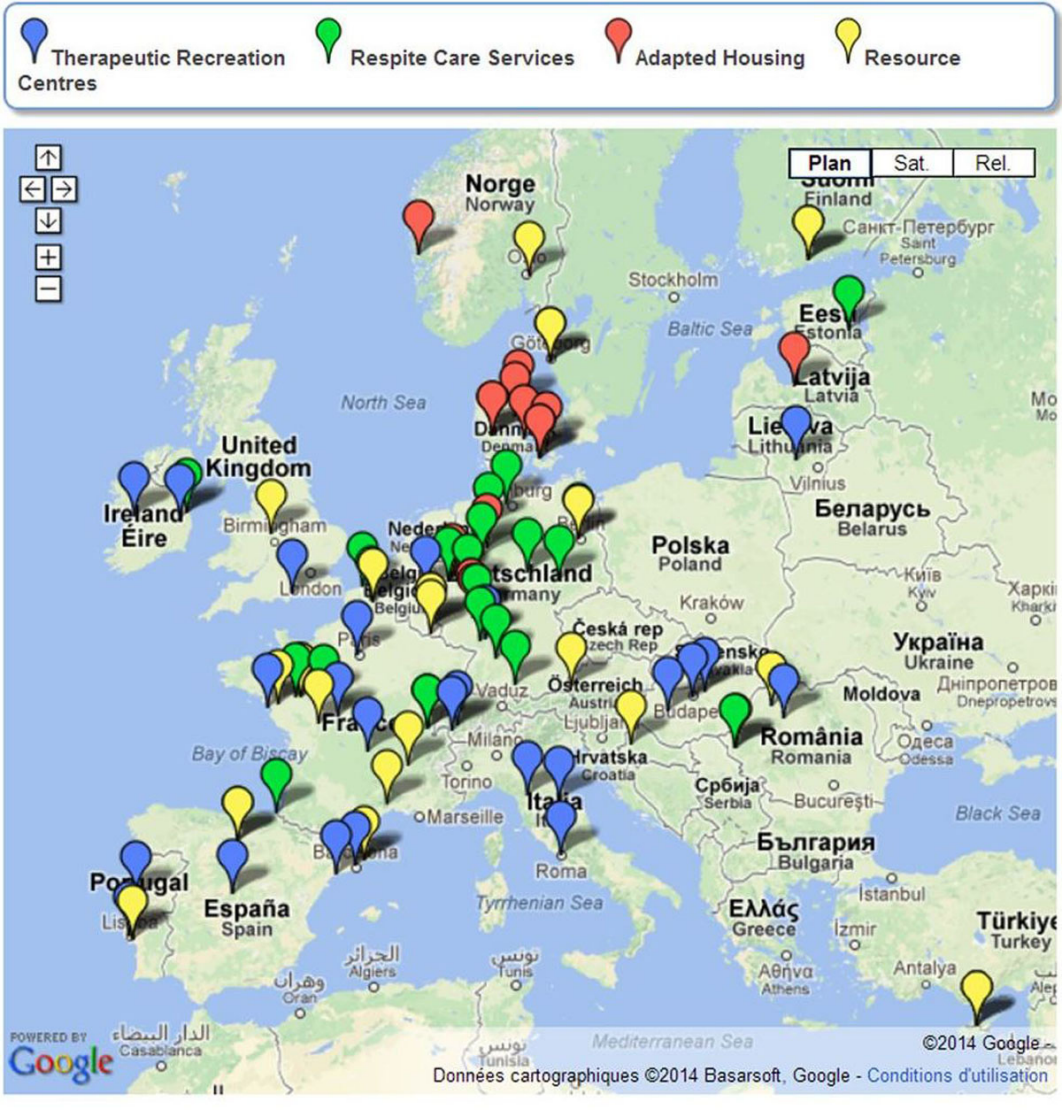
Map of Specialised Social Services.

The following results have been obtained: map of Specialised Social Services (Figure 1) and creation of EURODIS website section; Documents on ‘Guiding Principles for Specialised Social Services’ and ‘Training for Social Services Providers’; Case study documents on Agrenska (Sweden), Frambu (Norway), Bátor Tábor (Hungary) and Group Homes for Prader-Willi Syndrome (Denmark); policy fact sheets on Therapeutic Recreation Programmes, Respite Care Services, Adapted Housing Services, Resource Centres.

## Conclusion

European Committee of Experts on Rare Diseases Join Action Work Package 6 has now made available a set of information on specialised social services and on consensual good practices essential to the improvement of holistic care of people living with rare diseases. As the leader of this Joint Action Work Package, EURORDIS encourages decision-makers, patient representatives, national authorities, patients and families to use these tools to move forward in integrating people living with rare diseases into social services, in coordination with the national plans and strategies for rare diseases.

